# Reduced-Drift Virtual Gyro from an Array of Low-Cost Gyros

**DOI:** 10.3390/s17020352

**Published:** 2017-02-11

**Authors:** Richard J. Vaccaro, Ahmed S. Zaki

**Affiliations:** 1 Department of Electrical, Computer, and Biomedical Engineering, University of Rhode Island, Kingston, RI 02881, USA; 2 Naval Undersea Warfare Center, Division Newport, Newport, RI 02840, USA; ahmed.zaki@navy.mil

**Keywords:** virtual gyro, Allan variance, inertial sensor

## Abstract

A Kalman filter approach for combining the outputs of an array of high-drift gyros to obtain a virtual lower-drift gyro has been known in the literature for more than a decade. The success of this approach depends on the correlations of the random drift components of the individual gyros. However, no method of estimating these correlations has appeared in the literature. This paper presents an algorithm for obtaining the statistical model for an array of gyros, including the cross-correlations of the individual random drift components. In order to obtain this model, a new statistic, called the “Allan covariance” between two gyros, is introduced. The gyro array model can be used to obtain the Kalman filter-based (KFB) virtual gyro. Instead, we consider a virtual gyro obtained by taking a linear combination of individual gyro outputs. The gyro array model is used to calculate the optimal coefficients, as well as to derive a formula for the drift of the resulting virtual gyro. The drift formula for the optimal linear combination (OLC) virtual gyro is identical to that previously derived for the KFB virtual gyro. Thus, a Kalman filter is not necessary to obtain a minimum drift virtual gyro. The theoretical results of this paper are demonstrated using simulated as well as experimental data. In experimental results with a 28-gyro array, the OLC virtual gyro has a drift spectral density 40 times smaller than that obtained by taking the average of the gyro signals.

## 1. Introduction

The concept of combining the outputs of an array of high-drift sensors to produce a virtual low-drift sensor was introduced by Bayard and Ploen [1,2], who used a Kalman filter to produce the virtual sensor. They showed, through analysis and simulation examples, that the reduced drift of the virtual sensor depends on the drift components of the individual sensors being “favorably correlated” (i.e., having some negative correlation). In order to implement the Bayard approach it is necessary to estimate these correlations from sensor data, but no such estimation algorithm has appeared in the literature. Several researchers have attempted to implement the Bayard approach (see [3,4,5,6]). However, none of these efforts were able to implement the full Bayard algorithm because they were not able to estimate the correlations between the drift components of the individual sensors. In this paper we present an algorithm for estimating these correlations.

In addition to providing the Kalman filter algorithm for combining sensors, Bayard and Ploen also derived a mathematical formula for the drift of their optimal virtual sensor. This formula provides a lower bound on the drift of *any* method of combining the sensor outputs. In this paper we consider obtaining a virtual sensor by taking a linear combination of the individual sensor outputs. We derive a formula for computing the optimal coefficients and also derive a formula for the drift of the resulting virtual sensor. Our formula for the drift of the optimal linear combination virtual sensor is identical to Bayard and Ploen’s formula for the drift of the Kalman filter-based virtual sensor. Because the optimal drift can be obtained using only a linear combination of sensor outputs, we conclude that a Kalman filter is unnecessary for this problem.

The performance of a rate gyro or accelerometer is influenced by a number of factors including scale-factor errors, quantization effects, temperature effects, random drift, and additive noise. A comprehensive account of all of these factors for gyros is found in an IEEE Standard [7]. Among the many possible noise sources mentioned, the additive noise and random drift are often the dominant sources. In our experience, this is the case for tactical-grade MEMS sensors. Other researchers have made the same observation [8].

When an array of multiple sensors is considered, there may be correlations in the statistical properties of one sensor to another that can be exploited to reduce the drift. In this paper, we assume that the additive noise terms for different sensors are uncorrelated but that there may be correlations between the drift components of different sensors. This paper provides an algorithm to estimate these correlations from a finite amount of calibration data. We consider an array of gyros, but the modeling algorithm applies also to an array of accelerometers [9,10]. The organization of this paper, as well as its relationship to previous work, is as follows.

In Section 2.1 the standard single-gyro model for additive noise (ARW) and random drift (RRW) is introduced, along with the concept of Allan variance, which is computed in discrete-time using sampled gyro signals. The approach described here is based on the computed Allan variance of a single gyro, but is *not* based on the usual method of fitting lines to the Allan variance plot [7,11]. It was shown in [12] that this line-fitting method is a statistically inconsistent estimator. In [13] a modification of Allan variance using sliding windows was presented in order to produce a better plot. However, this modification is not necessary for the statistical modeling procedure used here because the procedure automatically deemphasizes inaccurate Allan variance points using theoretical formulas for the variance and covariance of computed Allan variance points. Other approaches to modeling individual gyros are presented in [14,15]. However, these approaches have not been extended to modeling the correlations in an array of gyros. In [16], the formulas of [10] were used to model triaxial rate gyros. However, the three gyro signals were assumed to be uncorrelated.

Section 2.4 presents a model for the signals in a two-gyro array, and introduces a new statistic that we call the *Allan covariance* between two gyros. The model for the correlation between two gyros can be extended to an array of many gyros by modeling all pairwise combinations of gyros. The statistical properties of a gyro array are described by the spectral density matrices, R and Q of the additive noise and random drift components of the gyro signals.

Section 2.7 extends to 2-gyro model to an array of gyros and derives an algorithm for estimating the off-diagonal elements of the Q matrix. Also derived is a formula for the coefficient vector $c^{*}$, which gives the optimal linear combination of gyro outputs to produce a scalar virtual gyro signal.

Section 2.9 presents simulation results for a 6-gyro array. Signals are generated using given Q and R matrices. Estimates $\hat{Q}$ and $\hat{R}$ are obtained from a finite amount of data using the algorithm from Section IV. The matrix $\hat{Q}$ matrix is used to calculate the coefficients for the optimal linear combination of gyro signals. The theoretical formula for the drift of the virtual gyro is confirmed by simulations.

Section 2.11 presents experimental results with an array of 28 MEMS gyros. The algorithm given in Section 2.7 is used to estimate the Q and R matrices for this gyro array and to compute the optimal coefficient vector $c^{*}$. The RRW spectral density of the resulting virtual gyro is calculated and found to be over 40-times smaller than that for a virtual gyro obtained by averaging the gyro outputs.

## 2. Results

### 2.1. Single-Gyro Model for Noise and Drift

The output signal, y(t), of a gyro in the absence of motion will be referred to as a *calibration signal*, and can be described by the following equations:

(1) $$\begin{array}{cc} y(t) & =b(t)+n(t)\\ \dot{b}\left(t\right) & =w(t) \end{array}$$

where b(t) is the bias (random drift), n(t) is observation noise, which is assumed to be white noise with spectral density *R*, and w(t) is another white-noise process described below in Equation (2). In the discrete-time treatment given below it is assumed that the gyro signal is lowpass filtered to a bandwidth *B* Hz. For rate gyros, the bias term b(t) is referred to as *rate random walk* or *RRW*, and the additive noise term n(t) is referred to as *angle random walk* or *ARW*.

The bias term b(t) is a random process consisting of the integral of a white noise process w(t) with spectral density *Q*. The integral creates a nonstationary random process whose variance increases with time, which provides a good model for the drift of a sensor. The bias signal is modeled as follows

(2) $$\dot{b}\left(t\right)=w\left(t\right),\;\;E\left[w\left(t_{1}\right)w\left(t_{2}\right)\right]=Q\delta \left(t_{1}-t_{2}\right).$$

The bias and noise terms are characterized by the numbers *Q* and *R*, respectively.

Allan variance, denoted a(τ), is computed for different values of a smoothing parameter, *τ*. In order to compute the Allan variance associated with a record of calibration data, the signal is partitioned into *N* *τ*-second intervals.

Let ${\bar{y}}_{k}$, k=1,⋯,N be the average values of the *N* nonoverlapping *τ*-second intervals of calibration data. Then the Allan variance statistic for this data set is defined as

(3) $$a\left(\tau \right)=\frac{1}{2(N-1)}\sum_{k=1}^{N-1}{\left({\bar{y}}_{k+1}-{\bar{y}}_{k}\right)}^{2}.$$

The sensor signal is sampled with a sampling interval of *T* seconds to obtain the discrete-time signal

(4) $$y_{k}=y\left(kT\right),\;k=1,\cdots ,N.$$

Consider the statistical characterization of the sampled noise and drift components of the sensor signal.

The noise term n(t) is modeled as bandlimited white noise with spectral density *R* and bandwidth *B* Hz. If the noise term is sampled with sampling interval T=1/(2B) second, the noise samples have variance R/T and noise samples at different sampling instants are uncorrelated. That is,

(5) $$E\left[n\left(k_{1}T\right)n\left(k_{2}T\right)\right]=\frac{R}{T}\delta \left[k_{1}-k_{2}\right],$$

where δ[k]=1 if k=0 and zero otherwise.

The drift term, b(t), is modeled as integrated white noise, as shown in Equation (2). The sampled bias term may be written as:

(6) $$y_{k}=b\left(kT\right)=\int_{0}^{kT}w\left(\tau \right)d\tau .$$

Let the random variables $z_{k}$, k=1,⋯,M, be the averages of *M* nonoverlapping length-*m* segments of data, where M=N/m:

(7) $$z_{1}=\frac{y_{1}+\cdots +y_{m}}{m},\;\cdots ,z_{M}=\frac{y_{(M-1)m+1}+\cdots +y_{mM}}{m}.$$

In this analysis, the Allan variance smoothing parameter, *τ*, is τ=mT.

The Allan variance statistic is computed from a finite record of sampled data as follows:

(8) $$a\left[m\right]=\frac{1}{2(M-1)}\sum_{k=1}^{M-1}{\left(z_{k+1}-z_{k}\right)}^{2}.$$

### 2.2. Mean and Covariance

The results in this subsection were derived in [10]. The mean values for the ARW and RRW components of a[m] are:

(9) $$\mathrm{ARW}:E\left(a\left[m\right]\right)=\frac{R}{mT},\;\;\mathrm{RRW}:E\left(a\left[m\right]\right)=\frac{Q(mT)}{3}.$$

Consider now the Allan variances computed from a data record of *N* samples at two values of *m*, say $m_{1}$ and $m_{2}$, where $m_{2}=p\cdot m_{1}$ for some integer p≥1. We require that $M_{1}$ and $M_{2}$ defined as

(10) $$M_{1}=\frac{N}{m_{1}},\;M_{2}=\frac{N}{m_{2}}$$

are both integers. For the ARW component, the covariance between $a[m_{1}]$ and $a[pm_{1}]$ is

(11) $$\mathrm{covar}\left(a\left[m_{1}\right],a\left[pm_{1}\right]\right)=\frac{3M_{2}-4}{\left(M_{1}-1\right)\left(M_{2}-1\right)p^{2}}\frac{R^{2}}{{\left(m_{1}T\right)}^{2}}.$$

For the RRW component, covar($a\left[m_{1}\right],a\left[pm_{1}\right])$ =

(12) $$\frac{\left[\left(12p^{3}-6p+3\right)M_{2}-2\left(6p^{3}-3p+2\right)\right]Q^{2}{\left(m_{1}T\right)}^{2}}{36\left(M_{1}-1\right)\left(M_{2}-1\right)p^{2}}.$$

In order to use Equations (11) and (12) for a set of Allan variance points, the values of *m* used to compute these points must all be integer multiples of each other. This is accomplished by using values of *m* that are powers of 2. Consider a set of points $m=\left[\begin{array}{cccc} 2 & 4 & \cdots & 2^{q} \end{array}\right]$ for some integer *q*. Let a[m] be the corresponding vector of Allan variance statistics. The covariance matrices of a[m] for the ARW and RRW terms are obtained, element by element, from Equations (11) and (12), respectively. We denote these covariance matrices as

(13) $$\mathrm{covar}\left(a\left[m\right]\right)=C_{R}\left(m,R\right)\;\mathrm{for}\;\mathrm{ARW}$$

(14) $$\mathrm{covar}\left(a\left[m\right]\right)=C_{Q}\left(m,Q\right)\;\mathrm{for}\;\mathrm{RRW}.$$

For example, the ij element of the matrix $C_{R}\left(m,R\right)$ is covar($a\left[m_{i}\right],a\left[m_{j}\right]$), where $m_{i}$ and $m_{j}$ are the *i*th and *j*th elements, respectively of m and Equation (11) is used to evaluate the covariance.

### 2.3. Algorithm for Estimating Q and R for a Single Gyro

The algorithm presented in this section is based on standard results in statistical estimation theory, as expressed by the following theorem describing the best linear unbiased estimator [17]. This estimator has the lowest variance of any linear unbiased estimator.

**Theorem** **1.** *Given*

x=Hθ+w,

*where x is an N×1 vector of observations, H is a known N×p matrix, θ is a p×1 vector of parameters to be estimated, and w is zero-mean random vector with covariance matrix C. Then the best linear unbiased estimator for θ is*

(15) $$\hat{\theta }={\left(H^{T}C^{-1}H\right)}^{-1}H^{T}C^{-1}x.$$

*The expected value of the estimator is the true parameter vector, θ, and the covariance matrix of the estimator is*

(16) $$C_{\hat{\theta }}={\left(H^{T}C^{-1}H\right)}^{-1}$$

This theorem was used in [10] to obtain an algorithm for estimating *Q* and *R* for a single gyro and is used in Section IV below to derive an algorithm for modeling an array of gyros. The single-gyro algorithm is summarized in Algorithm 1.
**Algorithm 1:** An algorithm for estimating individual gyro parameters *Q* and *R*. ©2012 IEEE, reprinted with permission from [10].1.Given a data record of *N* points of gyro calibration data let $J=J_{1}-3$, where $J_{1}$ is the integer part of $\log_{2}\left(N\right)$. Calculate the Allan variance statistic for $m={\left[\begin{array}{cccc} 2 & 4 & \cdots & 2^{J} \end{array}\right]}^{T}$.2.Let ${\hat{\tau }}_{0}=Tm_{0}$ be an estimate of the smoothing lag corresponding to the minimum value of the Allan variance. For example, choose $m_{0}$ to be the index from Step 1 that gives the smallest Allan variance.3.From the values of *m* calculated in Step 1, select $m_{1}={\left[\begin{array}{ccc} 2 & \cdots & 2^{N_{1}} \end{array}\right]}^{T}$, such that the maximum value satisfies $2^{N_{1}}<{\hat{\tau }}_{0}/\left(8T\right)$.4.Obtain a preliminary estimate of *R*
$${\hat{R}}_{0}={\left(H_{R}^{T}C_{R}^{-1}\left(m_{1},1\right)H_{R}\right)}^{-1}H_{R}^{T}C_{R}^{-1}\left(m_{1},1\right)a\left[m_{1}\right],$$
where $H_{R}={\left[\begin{array}{ccc} 1/(2T) & \cdots & 1/(2^{N_{1}}T) \end{array}\right]}^{T}$.5.Calculate a preliminary estimate of *Q*:
${\hat{Q}}_{0}=3{\hat{R}}_{0}/{\hat{\tau }}_{0}^{2}.$6.Let (see Equations (13) and (14)):
$$C=C_{R}\left(m,{\hat{R}}_{0}\right)+C_{Q}\left(m,{\hat{Q}}_{0}\right)$$
where m is obtained in Step 1.7.The estimates of *Q* and *R* are given by
$$\left[\begin{array}{c} \hat{Q}\\ \hat{R} \end{array}\right]={\left(H^{T}C^{-1}H\right)}^{-1}H^{T}C^{-1}a\left[m\right],$$
where
$$H=\left[\begin{array}{cc} (2T)/3 & 1/(2T)\\ \vdots & \vdots \\ (2^{J-2}T)/3 & 1/(2^{J-2}T) \end{array}\right].$$

### 2.4. Mathematical Model for a Two-Gyro Array

In order to model an array of gyros it suffices to jointly model two gyros by estimating the correlation properties between the two gyros, as well as their individual statistical properties. Once the procedure is developed for two gyros it can be used for an array of any number of gyros by modeling all pairwise combinations of gyros.

The output signal vector, y(t), of a two-gyro array can be described by the following equation

(17) y(t)=ω(t)+b(t)+n(t)

where ω(t) is a vector of true rate signals, b(t) is a vector of gyro biases, and n(t) is an observation noise vector. In the absence of motion, the true rate is zero and the gyro output signal consists of bias plus noise. The bias term b(t) is a vector of *rate random walks* or *RRW*, and the additive noise term n(t) is a vector of *angle random walks* or *ARW*.

The elements of the noise vector n(t) are modeled as statistically independent bandlimited white noise processes with spectral densities $R_{1}$ and $R_{2}$, respectively, and common bandwidth *B* Hz. The spectral density matrix of n(t) is

(18) $$R=\left[\begin{array}{cc} R_{1} & 0\\ 0 & R_{2} \end{array}\right].$$

Let w(t) be a vector white-noise process with spectral density matrix Q. That is

(19) $$E\left[w\left(t_{1}\right)w\left(t_{2}\right)\right]=Q\delta \left(t_{1}-t_{2}\right),\;Q=\left[\begin{array}{cc} Q_{11} & Q_{12}\\ Q_{12} & Q_{22} \end{array}\right].$$

The bias vector is a random walk arising from w(t):

(20) $$\dot{b}\left(t\right)=w\left(t\right).$$

Gyro calibration data, with ω(t)=0, consists of bias and noise terms. These terms are characterized by the matrices Q and R, respectively. Given a finite record of gyro calibration data, the problem at hand is to estimate the nonzero elements of Q and R. In Algorithm 1 of the previous section, an algorithm is given for modeling an individual gyro. This algorithm may be used separately for gyro 1 and gyro 2 to obtain estimates of $Q_{11}$ and $R_{1}$ from gyro 1 data, and $Q_{22}$ and $R_{2}$ from gyro 2 data. All that remains is to show how to estimate $Q_{12}$ using the data from gyros 1 and 2.

Consider uniformly sampling the two gyros with sampling interval *T*:

(21) $$y_{k}=y\left(kT\right),\;y_{k}=\left[\begin{array}{c} y_{k}^{1}\\ y_{k}^{2} \end{array}\right],\;k=1,\cdots ,N.$$

Similarly to Equation (7) for a single gyro, define the vectors $z_{k}$ to be the average of *M* nonoverlapping length-m segments of vector-value gyro data $y_{k}$, where M=N/m:

(22) $$z_{1}=\frac{y_{1}+\cdots +y_{m}}{m},\;\cdots ,z_{M}=\frac{y_{(M-1)m+1}+\cdots +y_{mM}}{m}.$$

We define the Allan covariance matrix for a smoothing interval of *m* samples to be

(23) $$A\left[m\right]=\frac{1}{2(M-1)}\sum_{k=1}^{M-1}\left(z_{k+1}-z_{k}\right){\left(z_{k+1}-z_{k}\right)}^{T},$$

where M=N/m. Equation (23) is a simple extension of the single-gyro Allan variance formula (8). To our knowledge, this paper is the first time Equation (23) has been used to model an array of correlated gyros. The diagonal elements of A[m] are the usual Allan variances for gyros 1 and 2, respectively. The off diagonal terms, which are equal, are the Allan covariance between gyro 1 and gyro 2. We denote the off-diagonal term of A[m] as c[m], which is given by

(24) $$c\left[m\right]=\frac{1}{2(M-1)}\sum_{k=1}^{M-1}\left(z_{k+1}\left(1\right)-z_{k}\left(1\right)\right)\left(z_{k+1}\left(2\right)-z_{k}\left(2\right)\right),$$

where $z_{k}\left(1\right)$ is the first element of $z_{k}$ containing signals from gyro 1, and $z_{k}\left(2\right)$ is the second element of $z_{k}$ containing signals from gyro 2. The terms in the summation defining c[m] may be written using the following vectors of signal differences, for k=1,⋯,M−1:

(25) $$x_{k}=\left[\begin{array}{c} y_{k\cdot m+1}^{1}-y_{(k-1)m+1}^{1}\\ \vdots \\ y_{(k+1)m}^{1}-y_{k\cdot m}^{1} \end{array}\right],w_{k}=\left[\begin{array}{c} y_{k\cdot m+1}^{2}-y_{(k-1)m+1}^{2}\\ \vdots \\ y_{(k+1)m}^{2}-y_{k\cdot m}^{2} \end{array}\right].$$

We can write

(26) $$z_{k+1}\left(1\right)-z_{k}\left(1\right)=\frac{1}{m}o^{T}x_{k},$$

where o is a vector of ones. Similarly, we can write

(27) $$z_{k+1}\left(2\right)-z_{k}\left(2\right)=\frac{1}{m}o^{T}w_{k}.$$

Then

(28) $$c\left[m\right]=\frac{1}{2m^{2}\left(M-1\right)}\sum_{k=1}^{M-1}o^{T}x_{k}w_{k}^{T}o.$$

### 2.5. Mean of the Allan Covariance

The mean of c[m] is found by calculating the expected value of $x_{k}w_{k}^{T}$ in Equation (28). The ARW components of the two gyros are statistically independent. Thus, $E[x_{k}w_{k}^{T}]=0$ and the mean of the ARW component of c[m] is zero.

In order to calculate the mean of the RRW component of c[m], define

(29) $${\tilde{R}}_{kk}=E\left[x_{k}w_{k}^{T}\right].$$

The *l*th elements of the vectors $x_{k}$ and $w_{k}$ are given by

(30) $$\begin{array}{c} x_{k}\left(l\right)=\int_{((k-1)m+l)T}^{(km+l)T}w_{1}\left(\tau_{1}\right)d\tau_{1},\\ w_{k}\left(l\right)=\int_{((k-1)m+l)T}^{(km+l)T}w_{2}\left(\tau_{2}\right)d\tau_{2}. \end{array}$$

Using these expressions, the ij element of the matrix ${\tilde{R}}_{kk}$ is calculated as follows:

(31) $$\begin{array}{c} E[x_{k}\left(i\right)w_{k}^{T}\left(j\right)]=\int_{((k-1)m+i)T}^{(km+i)T}\int_{((k-1)m+j)T}^{(km+k)T}E\left[w_{1}\left(\tau_{1}\right)w_{w}^{T}\left(\tau_{2}\right)\right]d\tau_{1}d\tau_{2}\\ =Q_{12}\int_{((k-1)m+i)T}^{(km+i)T}\int_{((k-1)m+j)T}^{(km+k)T}\delta \left(\tau_{1}-\tau_{2}\right)d\tau_{1}d\tau_{2}. \end{array}$$

The $\tau_{1}$ and $\tau_{2}$ integrals are each computed over intervals of length mT. The value of the double integral is computed by counting the number of length-*T* overlapping intervals in the $\tau_{1}$ and $\tau_{2}$ integrals. When i=j, the limits of integration overlap completely, giving a value of mT for the double integral. When j=i+1 or i−1 there are m−1 overlapping intervals and the value of the double integral is (m−1)T. In general, the number of overlapping intervals is m−|j−i|. Thus, for the RRW component, ${\tilde{R}}_{kk}$ is equal to

(32) $$\left[\begin{array}{ccccc} m & \left(m-1\right) & \cdots & 2 & 1\\ \left(m-1\right) & m & \ddots & 3 & 2\\ \vdots & \vdots & \ddots & \vdots & \vdots \\ 2 & 3 & \ddots & m & \left(m-1\right)\\ 1 & 2 & \cdots & \left(m-1\right) & m \end{array}\right]Q_{12}T.$$

Using Equation (28), the mean of c[m] is

(33) $$E\left(c\left[m\right]\right)=\frac{1}{2m^{2}\left(M-1\right)}\sum_{k=1}^{M-1}o^{T}{\tilde{R}}_{kk}o.$$

The term in the summation is simply the sum of all the elements of the matrix ${\tilde{R}}_{kk}$, which can be calculated using Equation (32) to be

(34) $$o^{T}{\tilde{R}}_{kk}o=\frac{2m^{3}+m}{3}Q_{12}T.$$

In practical applications the RRW term is significant only for large value of the smoothing parameter τ=mT, e.g., m>1000. Thus, only the highest power of *m* in Equation (34) is needed. Substituting Equation (34) into Equation (33) yields the mean of the RRW component of the Allan covariance:

(35) $$E\left(c\left[m\right]\right)=\frac{Q_{12}}{3}mT.$$

Because the mean of the ARW component of Allan variance is zero, the previous equation is also the mean of the Allan covariance of a gyro calibration signal.

### 2.6. Covariance of the Allan Covariance

The covariance between two values of the Allan covariance statistic, $c[m_{1}]$ and $c[m_{2}]$, is needed to obtain the best linear unbiased estimate of $Q_{12}$ using an algorithm similar to that used for modeling a single gyro. Let $m_{2}=p\cdot m_{1}$ for some integer p≥1. Let *N* be the number of samples in the sampled gyro calibration signals and let $M_{1}$ and $M_{2}$ be defined as

(36) $$M_{1}=\frac{N}{m_{1}},\;M_{2}=\frac{N}{m_{2}}.$$

By choosing $m_{1}$, $m_{2}$, and *N* to be powers of 2, both $M_{1}$ and $M_{2}$ will be integers. The covariance expressions for the ARW and RRW components are derived in the Appendix A. For the ARW component, the covariance is

(37) $$\mathrm{covar}\left(c\left[m_{1}\right],c\left[pm_{1}\right]\right)=\frac{3M_{2}-4}{2\left(M_{1}-1\right)\left(M_{2}-1\right)p^{2}}\frac{R_{1}R_{2}}{{\left(m_{1}T\right)}^{2}}.$$

Given a vector m containing *n* values of *m* (each a power of 2), let the vector a be the Allan covariance evaluated at each element of m. Then the n×n covariance matrix of a is calculated element-by-element using Equation (37). The resulting covariance matrix is called $C_{R}\left(m,R_{1},R_{2}\right)$.

For the RRW component, covar($\left(c\left[m_{1}\right],c\left[pm_{1}\right]\right)$ is given by the following expression:

(38) $$\frac{\left[\left(12p^{3}-6p+3\right)M_{2}-2\left(6p^{3}-3p+2\right)\right]\left(Q_{1}Q_{2}+Q_{12}^{2}\right){\left(m_{1}T\right)}^{2}}{72\left(M_{1}-1\right)\left(M_{2}-1\right)p^{2}}.$$

The n×n covariance matrix of a is calculated element-by-element using Equation (38). The resulting covariance matrix is called $C_{Q}\left(m,Q_{11},Q_{22},Q_{12}\right)$.

### 2.7. Statistical Model for a Gyro Array

Consider an array of *g* gyros. The Allan covariance matrix A[m] will be a g×g symmetric matrix. The off-diagonal terms, $A_{ij}\left[m\right]\;i\neq j$ are the Allan covariances between gyro *i* and gyro *j*. We assume that the ARW components of the different gyros are uncorrelated so that the off-diagonal terms of the spectral density matrix R are equal to zero. This uncorrelated assumption has been verified for the hardware gyro described in Section 2.11. Each of the diagonal elements of R may be estimated using the single-gyro algorithm given in Algorithm 1, which also gives the diagonal elements of the spectral density matrix Q. All that remains is to estimate the off-diagonal terms of Q, $Q_{ij}\;i\neq j$. An algorithm to do this is derived next.

The algorithm for estimating $Q_{ij}$ is based on Theorem 1. In order to use this theorem, let $m=\left[\begin{array}{cccc} 2 & 4 & \cdots & 2^{J} \end{array}\right]$ be a set of *J* smoothing indices and let a be the vector of corresponding Allan covariances

(39) $$a={\left[\begin{array}{cccc} A_{ij}\left[2\right] & A_{ij}\left[4\right] & \cdots & A_{ij}\left[2^{J}\right] \end{array}\right]}^{T}.$$

From Equation (34) the mean of a is

(40) $$E\left(a\right)=m\frac{T}{3}Q_{ij}.$$

The covariance of a is (see Equations (37) and (38))

(41) $$C=C_{R}\left(m,R_{i},R_{j}\right)+C_{Q}\left(m,Q_{i},Q_{j},Q_{ij}\right).$$

Knowing the mean and covariance of a, Theorem 1 may be used to obtain an estimator for $Q_{ij}$ as follows. Using the notation of Theorem 1, let x=a, H=mT/3, $\theta =Q_{ij}$, with C given by Equation (41). The only difficulty is that the matrix $C_{Q}$ in Equation (41) depends on $Q_{ij}$, the parameter we want to estimate. The simplest way to obtain a realizable algorithm is to set $Q_{ij}$ to zero in the formula for $C_{Q}$. The effect of this approximation will be examined in the simulation study in Section 2.9. The complete algorithm for estimating the off-diagonal entries of the matrix Q is given in Algorithm 2.
**Algorithm 2:** An algorithm for estimating the off-diagonal terms, $Q_{ij}$, of the spectral density matrix Q for the drift components of an array of gyros.1.Given a data record of *N* points of vector-valued gyro calibration data from an array of *g* gyros, let $J=J_{1}-3$, where $J_{1}$ is the integer part of $\log_{2}\left(N\right)$. Calculate the set of g×g Allan covariance matrices {A[m]} using Equations (21) through (23) for all m∈m, where $m={\left[\begin{array}{cccc} 2 & 4 & \cdots & 2^{J} \end{array}\right]}^{T}$.2.Estimate the individual gyro statistics ${\hat{R}}_{k}$ and ${\hat{Q}}_{kk},k=1,\cdots ,g$, using the algorithm for single gyros given in Algorithm 1.3.Let a be the vector whose elements are $A_{ij}\left[m\right]$ for all m∈m.4.Let
H=(T/3)m.5.Calculate the covariance matrix (see Equations (37) and (38))
$$C=C_{R}\left(m,{\hat{R}}_{i},{\hat{R}}_{j}\right)+C_{Q}\left(m,{\hat{Q}}_{i},{\hat{Q}}_{j},0\right)$$6.The estimate of $Q_{ij}$ is calculated as follows:
$${\hat{Q}}_{ij}={\left(H^{T}C^{-1}H\right)}^{-1}H^{T}C^{-1}a$$

### 2.8. Optimal Linear Combination of Gyro Signals

Suppose we have an array of *g* gyros with a known RRW matrix Q. The outputs of the gyros are collected into a vector y(t). A virtual gyro signal v(t) can be obtained by taking a fixed linear combination of the gyro signals:

(42) $$v\left(t\right)=c^{T}y\left(t\right)$$

where the coefficient vector $c={\left[\begin{array}{ccc} c_{1} & \cdots & c_{g} \end{array}\right]}^{T}$ satisfies

(43) $$o^{T}c=1,\;\mathrm{with}\;o={\left[\begin{array}{ccc} 1 & \cdots & 1 \end{array}\right]}^{T}.$$

Each gyro is measuring the same velocity signal ω(t). The constraint in Equation (43) implies that the weighted signal components in each of the gyro signals will add up to ω(t).

The signal v(t) can be modeled as the output of a single gyro with some effective RRW spectral density matrix $Q_{v}$. We define the optimal coefficient vector $c^{*}$ to be the one that results in the virtual gyro having the smallest value of $Q_{v}$.

From Equations (20) and (42), the bias signal for the virtual gyro satisfies

(44) $$\dot{v}\left(t\right)=c^{T}\dot{y}\left(t\right)=c^{T}w\left(t\right).$$

Comparing Equation (44) with Equation (2), it can be seen that the spectral density of the RRW component of the virtual gyro is

(45) $$\begin{array}{cc} E[\left(c^{T}w\left(t_{1}\right)\right)\left(c^{T}w\left(t_{2}\right)\right)]\; & \;=E[\left(c^{T}w\left(t_{1}\right)\right){\left(w\left(t_{2}\right)\right)}^{T}c]\\ \; & \;=c^{T}Qc\delta \left(t_{1}-t_{2}\right). \end{array}$$

Thus, $Q_{v}$ for a virtual gyro signal formed as a fixed linear combination of gyro signals is

(46) $$Q_{v}=c^{T}Qc.$$

The optimal coefficient vector $c^{*}$ is the one that minimizes the drift of the virtual gyro Equation (46) subject to the constraint in Equation (43). That is,

(47) $$c^{*}=\arg\min_{c}c^{T}Qc\;\mathrm{such}\;\mathrm{that}\;o^{T}c=1.$$

Using a Lagrange multiplier to convert this to an unconstrained optimization problem and setting the first derivative to zero yields the well-known solution to Equation (47):

(48) $$c^{*}=\frac{Q^{-1}o}{o^{T}Q^{-1}o}.$$

The virtual gyro signal obtained by taking the linear combination of gyro signals using the coefficient vector $c^{*}$ will have an RRW spectral density $Q_{v}^{*}$ given by Equation (46). Substituting Equation (48) in Equation (46) yields

(49) $$Q_{v}^{*}=\frac{1}{o^{T}Q^{-1}o}.$$

Because o is a vector of ones, the previous equation says that $Q_{v}^{*}$, the RRW spectral density of the optimal linear combination (OLC) virtual gyro, is equal to the reciprocal of the sum-of-elements of the matrix $Q^{-1}$. This is identical to the virtual gyro drift obtained from the Kalman filter approach. In column 10 of Bayard and Ploen’s patent [2] they say that “the theoretically minimum drift attainable from combining *N* gyro devices having Rate Random Walk matrix Q is given by 1/(sum of the elements of $Q^{-1})$.” Thus the minimum drift achieved by the Kalman filter based (KFB) approach is identical to that achieved by the OLC approach presented in this paper.

Consider the following similarities and differences between the KFB and OLC approaches. In both cases the algorithms are derived for gyros that are modeled using only ARW and RRW components. In both cases the matrix Q must be estimated from calibration data that is obtained while the array is motionless. In order to solve the Kalman filter equations the estimated matrix $\hat{Q}$ must be positive definite. For the OLC approach, the theoretical formulas assume a positive definite $\hat{Q}$ but it is shown in Equation (54) (and preceding text) how to handle the case when $\hat{Q}$ is not positive definite. Finally, to calculate the KFB virtual gyro output from an array of *g* gyros in an operational environment, the gyro signals must be processed by an *g*th-order Kalman filter. In contrast the OLC virtual gyro simply forms a weighted sum of the *g* gyro signals using a fixed linear combination.

### 2.9. Simulation Example

The example in this section consists of a simulation of an array of six gyros. The matrix R is a diagonal matrix with units of $\deg^{2}/(hr)$ and the following diagonal elements:

(50) $$10^{-3}\times \left[\begin{array}{cccccc} 0.1010 & 0.0712 & 0.0490 & 0.0383 & 0.0385 & 0.0914 \end{array}\right].$$

The matrix Q, with units $\deg^{2}{/(hr)}^{3}$, is

(51) $$\left[\begin{array}{cccccc} 0.0119 & -0.0004 & 0.0048 & -0.0076 & -0.0112 & -0.0026\\ -0.0004 & 0.0220 & 0.0093 & 0.0005 & 0.0015 & 0.0097\\ 0.0048 & 0.0093 & 0.1628 & -0.0598 & 0.0026 & -0.0141\\ -0.0076 & 0.0005 & -0.0598 & 0.0976 & 0.0191 & 0.0254\\ -0.0112 & 0.0015 & 0.0026 & 0.0191 & 0.0259 & 0.0114\\ -0.0026 & 0.0097 & -0.0141 & 0.0254 & 0.0114 & 0.1167 \end{array}\right].$$

We begin with some theoretical calculations based on the known Q matrix. Then, in the second subsection below, we present results using simulated gyro signals.

#### Theoretical Calculations

The drift quality of the individual gyros is given by the diagonal elements of Q. Gyro 3 is the worst, with $Q_{33}=163\times 10^{-3}$. Gyro 1 is the best, with $Q_{11}=11.9\times 10^{-3}$.

There are several ways that a virtual gyro signal can be obtained by taking a fixed linear combination of the outputs of the six gyros. One way is to take the average of the gyro signals using the coefficient vector $c={\left[\begin{array}{ccc} 1/6 & \cdots & 1/6 \end{array}\right]}^{T}$. Another way is to take a weighted average of the gyro signals in which the weights are the inverses of the *Q* values for each individual gyro. This is equivalent to using only the diagonal elements of the matrix Q in Equation (48). A third way is to use the optimal linear combination $c^{*}$ given by Equation (48). The RRW spectral density $Q_{v}$ for any virtual array created by taking a fixed linear combination of gyro signals can be computed using Equation (46). The results for the three methods just mentioned are shown in Table 1.

Several observations can be made about this example. First, the simple average of the gyro signals does not improve the drift of the best individual gyro, mostly because the individual gyros have very different *Q* values. This disparity in *Q* values is accounted for using only the diagonal elements of Q in the calculation of the coefficient vector, which greatly improves the value of $Q_{v}$. Finally, the optimal linear combination, which is calculated using the entire Q matrix, gives the lowest value of $Q_{v}$.

### 2.10. Simulation Results

**Figure 1:**
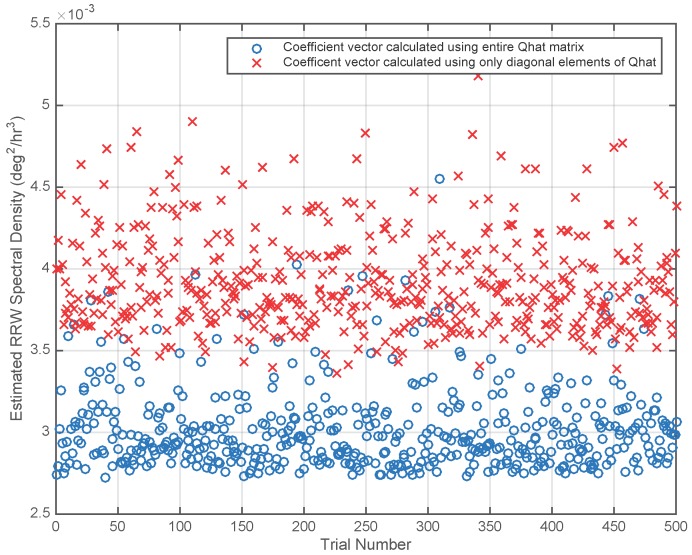
Random drift (RRW) spectral density ${\hat{Q}}_{v}$ for virtual gyros corresponding to coefficient vectors calculated using only the diagonal elements of $\hat{Q}$ and using the entire $\hat{Q}$ matrix. A virtual gyro created by averaging the gyro signals has an RRW spectral density of $11.5\times 10^{-3}$ for all trials (see Table 1).

In order to test Algorithm 2, 500 realizations of the gyro signals were created at 10 Hz sampling rate for a duration of 31.1 hours using the spectral density matrices given in Equations (50) and (51). For each of the 500 trials the matrices $\hat{Q}$ and $\hat{R}$ were computed using Algorithm 2. Two different coefficient vectors were computed. The first used the diagonal elements of $\hat{Q}$ in Equation (48). The second, optimal coefficient vector, was computed using the full $\hat{Q}$ matrix in Equation (48). Finally, virtual gyro signals were created by taking linear combinations of the six gyro signals using the two coefficient vectors. The virtual gyro signals were modeled using Algorithm 1 and the spectral densities $\left({\hat{R}}_{v},{\hat{Q}}_{v}\right)$ for the virtual gyros were recorded. Figure 1 is a plot of the ${\hat{Q}}_{v}$ values corresponding to the two coefficient vectors for each trial. For the virtual gyros created using only the diagonal elements of $\hat{Q}$, the resulting ${\hat{Q}}_{v}$ values had a mean of $3.9\times 10^{-3}$ and a standard deviation of $2.9\times 10^{-4}$. The mean of ${\hat{Q}}_{v}$ is 3% larger than the theoretical value shown in Table 1 under Method 2. The ${\hat{Q}}_{v}$ values for the virtual gyros created using the full $\hat{Q}$ matrix had a mean of $3.0\times 10^{-3}$ and a standard deviation of $2.5\times 10^{-4}$. This mean value is 11% larger than the theoretical value shown in Table 1 under Method 3 due to errors in estimating the the off-diagonal elements of Q. We believe that those errors are due primarily to the approximation used in computing $C_{Q}$. See Equation (41) and the discussion following.

### 2.11. Hardware Results for a 28-Gyro Array

The algorithm has been implemented on a sensor array developed by NUWC. The sensor array consisted of 28 3-axis MEMS gyroscopes mounted on a flat board. The gyroscope part number is L3GD20 from ST Microelectonics. Calibration data were collected while the gyro array was motionless (lying on a table) at room temperature. The outputs of each gyro were lowpass filtered and sampled at 10 Hz. Each pair of 14 sensors are handled by a Propeller multicore microcontroller. The XYZ data from each all the sensors are packaged, time tagged and streamed to the computer through a high-speed USB port in binary format. Dedicated software on the PC was used to log the data and convert to ASCII upon saving it to a file.

The gyro signals were recorded for 58.3 hours and the *x*-axis signals were analyzed. In order to test the assumption that the ARW components of different gyros are uncorrelated, the Algorithm 2 was modified to estimate the entire $\hat{R}$, including the off-diagonal elements, as well as the matrix $\hat{Q}$. The diagonal elements of $\hat{R}$ were sorted and compared with the eigenvalues of $\hat{R}$, which were also sorted. The entries in the two lists varied only in the third significant figures, indicating that the ARW components are indeed uncorrelated. The remainder of this section uses the Algorithm 2, which assumes that R is a diagonal matrix. By not explicitly estimating the off-diagonal terms in R there are fewer parameters to estimate, which results in a more accurate estimate of the off-diagonal elements of Q.

**Figure 2:**
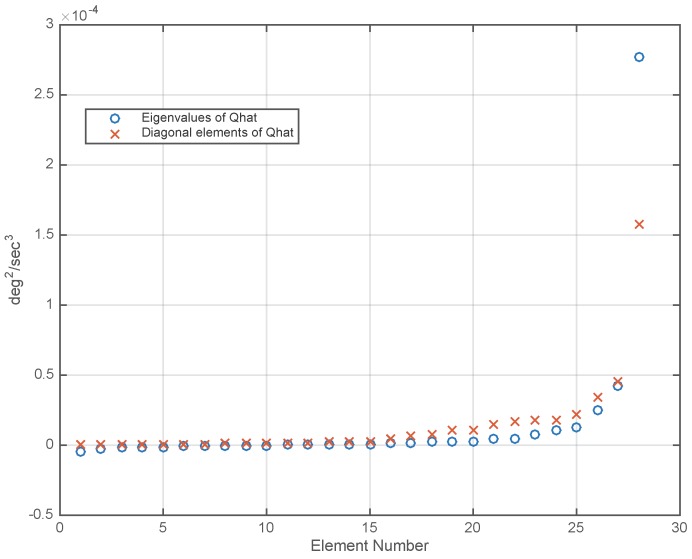
Sorted eigenvalues and sorted diagonal elements of the matrix $\hat{Q}$, which was estimated from data from a 28-gyro array.

The sorted eigenvalues of $\hat{Q}$ and the sorted diagonal elements of $\hat{Q}$ are plotted in Figure 2. The differences in the elements of these two lists indicates correlation between the RRW components of the gyros, which can be used for drift reduction.

However, Figure 2 also reveals a difficulty that must be addressed before computing the optimal linear combination vector $c^{*}$ from $\hat{Q}$. The difficulty is that $\hat{Q}$ has several negative eigenvalues, which are due to errors in estimating the individual entries in the $\hat{Q}$ matrix. If $\hat{Q}$ has negative eigenvalues then the quantity $c^{T}\hat{Q}c$, which is minimized to obtain the optimal linear combination coefficient vector, may take negative values. In this case, the result of the minimization will be the largest possible negative value of $c^{T}\hat{Q}c$, which is not a useful result. There are several ways to deal with this difficulty, and we have found the following to be the most effective. Let the singular value decomposition (SVD) of $\hat{Q}$ be written as follows:

(52) $$\hat{Q}=\sum_{k=1}^{g}s_{k}u_{k}v_{k}^{T}$$

where $u_{k}$ and $v_{k}$ are the left and right singular vectors, respectively, of $\hat{Q}$, and $s_{k}$ are the singular values. The inverse of $\hat{Q}$ may be written as:

(53) $${\hat{Q}}^{-1}=\sum_{k=1}^{g}s_{k}^{-1}u_{k}v_{k}^{T}.$$

When $\hat{Q}$ has negative eigenvalues, we propose to compute a partial inverse by omitting the first term or two in the SVD expansion of ${\hat{Q}}^{-1}$. These are the least important terms because they contain the inverse of the largest singular values. For the $\hat{Q}$ obtained from the 28-gyro array only the first term was omitted, and the coefficients for the optimal linear combination were computed as follows, using x as a temporary variable (see Equation (48)):

(54) $$x=\sum_{k=2}^{28}s_{k}^{-1}u_{k}v_{k}^{T};\;\;\;c^{*}=\frac{x}{o^{T}x}.$$

**Figure 3:**
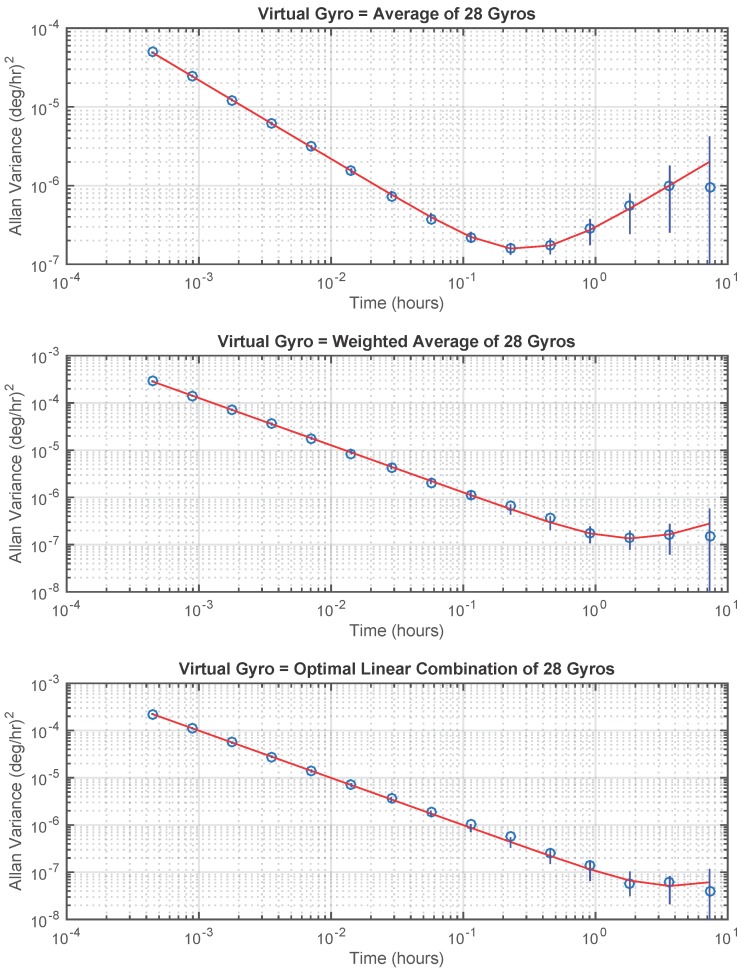
Allan variance plots for virtual gyros. Circles are calculated Allan variance points. Smooth curves are theoretical Allan variance plots corresponding to the $\left(\hat{R},\hat{Q}\right)$ obtained for each virtual gyro using Algorithm 1. Vertical lines through each point are ±2 standard deviations calculated using $\left(\hat{R},\hat{Q}\right)$. (**Top plot**): average of 28 gyros. (**Middle plot**): weighted average of 28 gyros using only diagonal elements of $\hat{Q}$. (**Lower plot**): optimal linear combination of 28 gyros.

A virtual gyro signal was created by taking a linear combination of the 28 gyro signals using three different methods. Method 1 is the unweighted average of the gyro signals. Method 2 uses only the diagonal elements of $\hat{Q}$ in Equation (48) to calculate the coefficients. Method 3 is the optimal linear combination, which is calculated using Equation (54). The Allan variance plots for these three virtual gyros are shown in Figure 3 and the estimated *R* and *Q* parameters are shown in Table 2.

Table 2 shows that, for this set of experimental data, using only the diagonal elements of $\hat{Q}$ gives a virtual gyro with a value of ${\hat{Q}}_{v}$ that is a factor of 8-times smaller than that obtained by an unweighted average of the gyro signals. Using the full $\hat{Q}$ matrix (with partial inverse) decreases ${\hat{Q}}_{v}$ by an additional factor of 5.

Note that if the RRW components of the 28 gyros in the array were uncorrelated, the theoretical matrix Q would be a diagonal matrix. Due to estimation errors, the matrix $\hat{Q}$ would contain nonzero off-diagonal terms, but these off-diagonal terms would not contain any useful information. The fact that the linear combination obtained using the full $\hat{Q}$ matrix gave substantially better results than the linear combination obtained using only the diagonal elements of $\hat{Q}$ is evidence that the off-diagonal terms of the theoretical Q matrix are nonzero. That is, there is evidence that the RRW components for the gyros in this hardware array are correlated.

## 3. Discussion and Conclusions

We have presented an algorithm for estimating R and Q, the spectral density matrices for the ARW and RRW components, respectively, of an array of gyros. The algorithm is based on the definition of the Allan covariance between two gyros, which is a generalization of the Allan variance for a single gyro. We considered virtual gyros, defined by fixed linear combinations of the signals from a gyro array, and derived a formula for the coefficients of the optimal linear combination resulting in the virtual gyro with smallest drift.

The performance of the minimum-drift virtual gyro was demonstrated using simulated as well as experimental data. Experimental results with an array of 28 gyros showed that the proposed virtual gyro had a drift spectral density 40-times smaller than that obtained by taking the average of the gyro signals.

One area of future work is to extend the approach presented in this paper to handle gyros who calibration signals are modeled by more than just ARW and RRW components. For example, references [14,15] show how to model a single gyro which has ARW, RRW, and Gauss-Markov components. Although all of these components are needed to accurately model the gyro, the long-term drift of a gyro is determined only by the RRW component. If the modeling procedure in [14,15], or some other modeling procedure, were extended to a gyro array, only the spectral density matrix Q of the RRW components would be needed to obtain a minimum-drift OCL virtual gyro. The optimal coefficients would still be calculated using Equation (48).

In order for the minimum-drift gyro to be much better than the average of the gyros, the off-diagonal entries in the matrix Q must be nonzero; that is, there must be correlations among the RRW components of different gyros in the array. Another important area of future work is to determine what aspects of gyro fabrication influence the correlation properties between gyros. Finally, we mention that other approaches to combining multiple gyros and accelerometers are presented in [18]. It is possible that the statistical analysis presented above could be combined with the ideas in [18].

## Figures and Tables

**Table 1:** Coefficient vectors for a 6-gyro array and corresponding RRW spectral density $Q_{v}$ using three methods. Method 1 is the unweighted average of the gyro signals. Method 2 uses only the diagonal elements of the theoretical Q matrix to calculate the coefficients. Method 3 is the optimal linear combination, which is calculated using the entire Q matrix.

	Method 1	Method 2	Method 3
Coefficient Vector	$\left[\begin{array}{c} 0.1667\\ 0.1667\\ 0.1667\\ 0.1667\\ 0.1667\\ 0.1667 \end{array}\right]$	$\left[\begin{array}{c} 0.4353\\ 0.2354\\ 0.0318\\ 0.0531\\ 0.2000\\ 0.0444 \end{array}\right]$	$\left[\begin{array}{c} 0.5600\\ 0.1196\\ -0.0145\\ -0.0039\\ 0.3480\\ -0.0092 \end{array}\right]$
$Q_{v}$	$11.5\times 10^{-3}$	$3.8\times 10^{-3}$	$2.7\times 10^{-3}$

**Table 2:** Estimated values of ARW and RRW spectral densities for virtual gyros obtained from experimental data by three different methods. Method 1 is the unweighted average of the gyro signals. Method 2 uses only the diagonal elements of $\hat{Q}$ to calculate the coefficients. Method 3 is the optimal linear combination, which is calculated using the entire $\hat{Q}$ matrix.

Method	ARW	RRW
1	$2.2\times 10^{-8}$	$82.3\times 10^{-8}$
2	$12.7\times 10^{-8}$	$10.8\times 10^{-8}$
3	$9.9\times 10^{-8}$	$2.0\times 10^{-8}$

## Appendix A. Derivation of Covariance of Allan Covariance Between Two Gyros

Consider the second moment of the Allan covariance c[m] defined in Equation (28) .

(A1) $$E(c^{2}\left[m\right])=\frac{1}{4m^{4}{\left(M-1\right)}^{2}}\sum_{i=1}^{M-1}\sum_{j=1}^{M-1}E\left[\left(o^{T}x_{i}\right)\left(o^{T}w_{i}\right)\left(o^{T}x_{j}\right)\left(o^{T}w_{j}\right)\right].$$

The expectation inside the double summation contains the product of four Gaussian random variables. Consider the following formula for such an expectation [19,20]:

(A2) $$\begin{array}{c} E\left(v_{1}v_{2}v_{3}v_{4}\right)\;=\;E\left(v_{1}v_{2}\right)E\left(v_{3}v_{4}\right)+E\left(v_{1}v_{3}\right)E\left(v_{2}v_{4}\right)\\ \;+\;E\left(v_{1}v_{4}\right)E\left(v_{2}v_{3}\right)-2E\left(v_{1}\right)E\left(v_{2}\right)E\left(v_{3}\right)E\left(v_{4}\right). \end{array}$$

The correspondence between Equations (A1) and (A2) is as follows:

(A3) $$v_{1}=\left(o^{T}x_{i}\right),\;v_{2}=\left(o^{T}w_{i}\right),\;v_{3}=\left(o^{T}x_{j}\right),\;v_{4}=\left(o^{T}w_{j}\right).$$

Each of the random variables in Equation (A3) has zero mean, thus the last term of Equation (A2) is equal to zero. The first term of Equation (A2) , using Equations (A3) and (29), is

(A4) $$E\left(\left(o^{T}x_{i}\right)\left(o^{T}w_{i}\right)\right)E\left(\left(o^{T}x_{j}\right)\left(o^{T}w_{j}\right)\right)=\left(o^{T}{\tilde{R}}_{ii}o\right)\left(o^{T}{\tilde{R}}_{jj}o\right).$$

Substituting this into the right-hand side of Equation (A1) and using Equation (33) yields $E^{2}\left(c\left[m\right]\right)$. This square of the mean can be subtracted from $E(c^{2}\left[m\right])$ to yield the variance of c[m]. Thus, the second and third terms of the right-hand side of Equation (A2), substituted into Equation (A1), give the variance of c[m]. These two terms will be called “term 2” and “term 3,” respectively. They are given by

(A5) $$\mathrm{term}\;2=\frac{1}{4m^{4}{\left(M-1\right)}^{2}}\sum_{i=1}^{M-1}\sum_{j=1}^{M-1}\left(o^{T}R_{ij}^{1}o\right)\left(o^{T}R_{ij}^{2}o\right)$$

and

(A6) $$\mathrm{term}\;3=\frac{1}{4m^{4}{\left(M-1\right)}^{2}}\sum_{i=1}^{M-1}\sum_{j=1}^{M-1}\left(o^{T}{\tilde{R}}_{ij}o\right)\left(o^{T}{\tilde{R}}_{ij}^{T}o\right),$$

where

(A7) $${\tilde{R}}_{ij}=E\left[x_{i}w_{j}^{T}\right]$$

and $R_{ij}^{1}$ and $R_{ij}^{2}$ are defined as follows:

(A8) $$R_{ij}^{1}=E\left(x_{i}x_{j}^{T}\right)\;(\mathrm{gyro}\;1),\;\;R_{ij}^{2}=E\left(w_{i}w_{j}^{T}\right)\;(\mathrm{gyro}\;2).$$

The variance of c[m] is the sum of terms 2 and 3, and the values of these terms are computed next.

The following single-gyro formulas for the variance of the Allan variance a[m] (see Equation (8)) were derived in [10].

(A9) $$\mathrm{var}\left(a\left[m\right]\right)=\frac{1}{2m^{4}{\left(M-1\right)}^{2}}\sum_{i=1}^{M-1}\sum_{j=1}^{M-1}{\left(o^{T}R_{ij}o\right)}^{2},$$

where

(A10) $$R_{ij}=E\left(x_{i}x_{j}\right).$$

For the ARW component,

(A11) $$\mathrm{var}\left(a\left[m\right]\right)=\frac{3M-4}{{\left(M-1\right)}^{2}}\frac{R^{2}}{{\left(mT\right)}^{2}}.$$

For the RRW component,

(A12) $$\mathrm{var}\left(a\left[m\right]\right)=\frac{9M-10}{36{\left(M-1\right)}^{2}}Q^{2}{\left(mT\right)}^{2}.$$

Term 2 in Equation (A5) may be compared with the formula for a single gyro given by Equation (A9). Equation (A5) has an extra factor of 2 in the denominator and has the product of two different gyros, $\left(o^{T}R_{ij}^{1}o\right)\left(o^{T}R_{ij}^{2}o\right)$ instead of the square of the term for one gyro. Thus, term 2 is given by the variance formulas (A11) and Equation (A12) with the following changes: $R^{2}$ is replaced by $R_{1}R_{2}$ in Equation (A11) , $Q^{2}$ is replaced by $Q_{1}Q_{2}$ in Equation (A12), and both expressions are divided by 2. The results are as follows:

(A13) $$\begin{array}{cc} \mathrm{ARW}:\mathrm{term}\;2\; & \;=\frac{3M-4}{2{\left(M-1\right)}^{2}}\frac{R_{1}R_{2}}{{\left(mT\right)}^{2}}\\ \mathrm{RRW}:\mathrm{term}2\; & \;=\frac{9M-10}{72{\left(M-1\right)}^{2}}Q_{1}Q_{2}{\left(mT\right)}^{2}. \end{array}$$

Term 3 in Equation (A6) for ARW is zero because additive noises on different gyros are uncorrelated. In order to compute term 3 for RRW, note that the sum of elements of ${\tilde{R}}_{ij}$ is equal to the sum of elements of ${\tilde{R}}_{ij}^{T}$. Thus, the summand of Equation (A6) may be written as ${\left(o^{T}{\tilde{R}}_{ij}o\right)}^{2}$. Now compare the definition of ${\tilde{R}}_{ij}$ in Equation (A7) with the definition of $R_{ij}$ in (A10). In Equation (A10) the expectation is taken of a product of signal difference vectors from a single gyro. When the data in the signal difference vectors come from overlapping intervals, the expectation is *Q* times the length of the overlap. In Equation (A7) the expectation is taken of the product of signal difference vectors from two different gyros. When the data in the signal difference vectors come from overlapping intervals the expectation is $Q_{12}$ times the length of the overlap. The intervals in Equation (A9) are the same lengths as those in Equation (A7). The expected values are squared in Equation (A9) as well as in Equation (A6). Thus, term 3 for RRW is given by the variance formula (A12) with the following changes: $Q^{2}$ is replaced by $Q_{12}^{2}$ and the expression is divided by 2. The results are as follows:

(A14) $$\begin{array}{cc} \mathrm{ARW}:\mathrm{term}\;3\; & \;=0\\ \mathrm{RRW}:\mathrm{term}3\; & \;=\frac{9M-10}{72{\left(M-1\right)}^{2}}Q_{12}^{2}{\left(mT\right)}^{2}. \end{array}$$

Summing terms 2 and 3 using Equations (A13) and (A14) yields the ARW and RRW variance expressions for the Allan covariance c[m].

To summarize, the ARW and RRW formulas for var(c[m]) given by summing terms 2 and 3 in Equation (A13) and (A14) are identical to the corresponding formulas (A11) and (A12) for var(a[m]) when the following substitutions are made in the latter formulas:

(A15) $$\begin{array}{c} \mathrm{ARW}:R^{2}\;\mathrm{is}\;\mathrm{replaced}\;\mathrm{by}R_{1}R_{2}/2\\ \mathrm{RRW}:Q^{2}\;\mathrm{is}\;\mathrm{replaced}\;\mathrm{by}\;\left(Q_{1}Q_{2}+Q_{12}^{2}\right)/2. \end{array}$$

Consider now the covariance between two values of the Allan covariance, $c[m_{1}]$ and $c[m_{2}]$, where $m_{2}=p\cdot m_{1}$ for some integer p≥1. The covariance calculations are similar to the variance calculations given above except that *m* is replaced by $m_{1}$ for the first gyro and $m_{2}$ for the second gyro. In [10] the same intervals of length $m_{1}$ and $m_{2}$ were used with data from a single gyro. As was seen in the variance calculations, the formulas for a single gyro may be used to get the results for two gyros using the substitutions shown in Equation (A15). Because the lengths of the overlapping intervals depend on $m_{2}=p\cdot m_{1}$ and are the same for the single gyro and two gyro cases, the expressions for covar($c\left[m_{1}\right],c\left[p\cdot m_{1}\right]$) may be obtained from Equations (11) and (12) by making the substitutions described in Equation (A15). The results are shown in Equations (37) and (38).

## References

[1] Bayard D.S., Ploen S.R. (2002). Foundations of Virtual Gyroscope Synthesis.

[2] Bayard D.S., Ploen S.R. (2005). High Accuracy Inertial Sensors From Inexpensive Components. U.S. Patent.

[3] Chang H., Xue L., Qin W., Yuan G., Yuan W. (2008). An integrated MEMS gyroscope array with higher accuracy output. Sensors.

[4] Totu L., Koldbaek S.K. (2011). Improving MEMS Gyroscope Performance Using Homogeneous Sensor Fusion. Master’s Thesis.

[5] Chang H., Xue L., Jiang C., Kraft M., Yuan W. (2012). Combining Numerous Uncorrelated MEMS Gyroscopes for Accuracy Improvement Based on an Optimal Kalman Filter. IEEE Trans. Instrum. Meas..

[6] Mallik H., Jyothi D., Varma M., Rao D., Agrawal V.K. Minimum Variance Optimal Filter Design for a 3 × 3 Gyroscope Cluster Configuration. Proceedings of the Fourth International Conference on Advances in Control and Optimization of Dynamical Systems.

[7] (1998). IEEE Standard Specification Format Guide and Test Procedure for Single-Axis Interferometric Fiber Optic Gyros.

[8] Ford J.J., Evans M.E. (2000). Online estimation of Allan variance parameters. J. Guid. Control Dyn..

[9] Vaccaro R.J., Zaki A.S. Statistical Modeling of Rate Gyros and Accelerometers. Proceedings of the 2012 IEEE/ION Position, Location and Navigation Symposium (PLANS 2012).

[10] Vaccaro R.J., Zaki A.S. (2012). Statistical Modeling of Rate Gyros. IEEE Trans. Instrum. Meas..

[11] El-Sheimy N., Hou H., Niu X. (2008). Analysis and modeling of inertial sensors using Allan variance. IEEE Trans. Instrum. Meas..

[12] Guerrier S., Molinari R., Stebler Y. (2016). Theoretical Limitations of Allan Variance-based Regression for Time Series Model Estimation. IEEE Signal Process. Lett..

[13] Li J., Fang J. (2013). Sliding Average Allan Variance for Inertial Sensor Stochastic Error Analysis. IEEE Trans. Instrum. Meas..

[14] Guerrier S., Skaloud J., Stebler Y., Victoria-Feser M. (2013). Wavelet-Variance-Based Estimation for Composite Stochastic Processes. J. Am. Stat. Assoc..

[15] Stebler Y., Guerrier S., Skaloud J., Victoria-Feser M. (2014). Generalized Method of Wavelet Moments for Inertial Navigation Filter Design. IEEE Trans. Aerosp. Electron. Syst..

[16] Yuan D., Ma X., Shang Z., Yan S. (2016). Statistical Modeling of Random Walk Errors for Triaxial Rate Gyros. IEEE Trans. Instrum. Meas..

[17] Kay S. (1993). Fundamentals of Statistical Signal Processing: Estimation Theory.

[18] Colomina I., Gimenez M., Rosales J.J., Wis M., Gómez A., Miguelsanz P. Redundant IMUs for Precise Trajectory Determination. Proceedings of the XXth ISPRS Congress.

[19] Janssen P.H.M., Stoica P. (1988). On the expectation of the product of four matrix-valued Gaussian random variables. IEEE Trans. Autom. Control.

[20] Bendat J.S., Piersol A.G. (2010). Random Data Analysis and Measurement Procedures.

